# Effects of chronic inhibition of Testosterone metabolism on cardiac remodeling after ischemia/reperfusion-induced myocardial damage in gonadectomized rats

**DOI:** 10.1242/bio.041905

**Published:** 2019-05-15

**Authors:** Octavio Maldonado, Angel Ramos, Mario Guapillo, Jose Rivera, Icela Palma, Ivan Rubio-Gayosso, Israel Ramirez-Sanchez, Nayelli Najera, Guillermo Ceballos, Enrique Mendez-Bolaina

**Affiliations:** 1 CIB-Doctorado en Ciencias Biomédicas-UV. Centro de Investigaciones Biomédicas, Universidad Veracruzana, Av. Dr. Luis Castelazo Ayala s/n, Colonia Industrial Anima, CP. 91000, Xalapa, Veracruz, Mexico; 2 Departamento de Nanotecnología, Universidad Tecnológica del Centro de Veracruz, Av. Universidad No. 350, Carretera Federal Cuitláhuac – La Tinaja, Localidad Dos Caminos, CP. 94910, Cuitláhuac, Veracruz, Mexico; 3 MCPB-Facultad de Ciencias Químicas, Universidad Veracruzana, Prolongación Oriente 6, No. 1009, Colonia Rafael Alvarado, CP. 94340, Orizaba, Veracruz, Mexico; 4 Laboratorio de Biología Molecular, Facultad de Ciencias Químicas, Universidad Veracruzana, Prolongación Oriente 6, No. 1009, Colonia Rafael Alvarado, CP. 94340, Orizaba, Veracruz, Mexico; 5 Seccion de Estudios de posgrado, Escuela Superior de Medicina, Instituto Politecnico Nacional, Plan de San Luis y Díaz Mirón, CP. 11340, Ciudad de México, Mexico

**Keywords:** Testosterone, 17β-estradiol, Cardiac remodeling, Metalloproteinase, Cardio-protection

## Abstract

The effects of testosterone on cardiovascular homeostasis are still not well understood. The objective of this work was to evaluate the effects of testosterone in the absence or presence of inhibition of Aromatase (4-hydroxyandrostenedione) and/or 5α reductase (Finasteride) enzymatic activities on the myocardial remodeling 30 days after ischemia/reperfusion (I/R) injury in gonadectomized rats. Results showed that testosterone administration to ORX rats resulted in decreased myocardial damaged area, inflammatory infiltrates and reduced MMP-3 and 13 expressions. Interestingly, Finasteride administration resulted in a greater decrease in scar tissue, inflammatory infiltrates, along with a significant decrease in MMP-3 and 13 expressions. In contrast, 4-hydroxyandrostenedione administrations increased all parameters. Our results suggest that testosterone does not have a direct effect since simultaneous inhibition of aromatase and 5α-reductase did not induce significant changes in I/R induced myocardial injury.

## Abstract (precis)

**Summary:** Coronary ischemia/reperfusion-induced injury in gonadectomyzed male rats is decreased by testosterone, protection is increased by blocking its 5α-reduction and blocked by inhibition of its aromatization.

## INTRODUCTION

Clinical studies have shown gender differences in the incidence of cardiovascular diseases. Men are more susceptible to developing cardiopathies than women who experience coronary heart disease and myocardial infarction usually 10 years later than men (Parks and Howlett, 2013; Czubryt et al., 2006; Booth and Lucchesi, 2008; Luczak and Leinwand, 2009; Ostadal et al., 2009; Regitz-Zagrosek and Seeland, 2012).

The effects of testosterone on cardiovascular homeostasis, however, are controversial. It is thought that testosterone increases the possibility of suffering ischemic heart disease in men (Araujo et al., 2007; Van der Wall, 2011). High doses of androgenic steroid supplementation accelerate atheroma progression increasing the risk of myocardial infarction and cerebrovascular events (Parker and Thompson, 2010; Phillips et al., 1994). Yet, there is no convincing evidence that physiological concentrations of testosterone have an impact on the development of ischemic heart disease (Carson and Rosano, 2012). In contrast, clinical studies have shown beneficial effects of testosterone on the cardiovascular system. It has been shown in long-term epidemiological studies that testosterone supplementation has a protective effect, reducing major cardiovascular events and mortality (Jones and Kelly, 2018). Accordingly, population studies have shown a strong relationship between decreased testosterone levels and increased cases of cardiovascular mortality (Ponikowska et al., 2010; Malkin et al., 2010).

Testosterone is converted into dihydrotestosterone (DHT) and 17β-estradiol by the action of the enzymes 5α-reductase and aromatase cytochrome P450 (CYP19), respectively (Czakja and Simpson, 2010). Thus, the contrasting findings mentioned above could be the result of an indirect effect of testosterone driven by its transformation into DHT or 17β-estradiol.

We had shown that administration of testosterone 15 min prior to reperfusion induced no changes in ischemia/reperfusion-induced (I/R) myocardial damage (after 4 h of reperfusion) in intact male rats, meanwhile, its administration protects the myocardium against ischemia/reperfusion damage in gonadectomized rats (Rubio-Gayosso et al., 2013). It also has been shown that testosterone supplementation in gonadectomized rats improved oxidative stress and decreased triglyceride accumulation (Regouat et al., 2018).

We also showed that testosterone metabolism into 17β-estradiol and/or DHT plays an important role in the testosterone-induced effects in gonadectomized rats.

We wonder if the chronic administration of testosterone in orchidectomized (ORX) rats modifies cardiac remodeling after 30 days of I/R-induced myocardial damage.

In this work we evaluated myocardial remodeling, inflammatory infiltrate and matrix metallopeptidase (MMP)-3 and MMP-13 expression in the absence and presence of inhibitors of testosterone 5α reduction or aromatization.

## RESULTS AND DISCUSSION

### Effect of testosterone supplementation, reductase and aromatase inhibition on myocardial damage induced by coronary I/R in orchidectomized rats

**Fig. 1.**
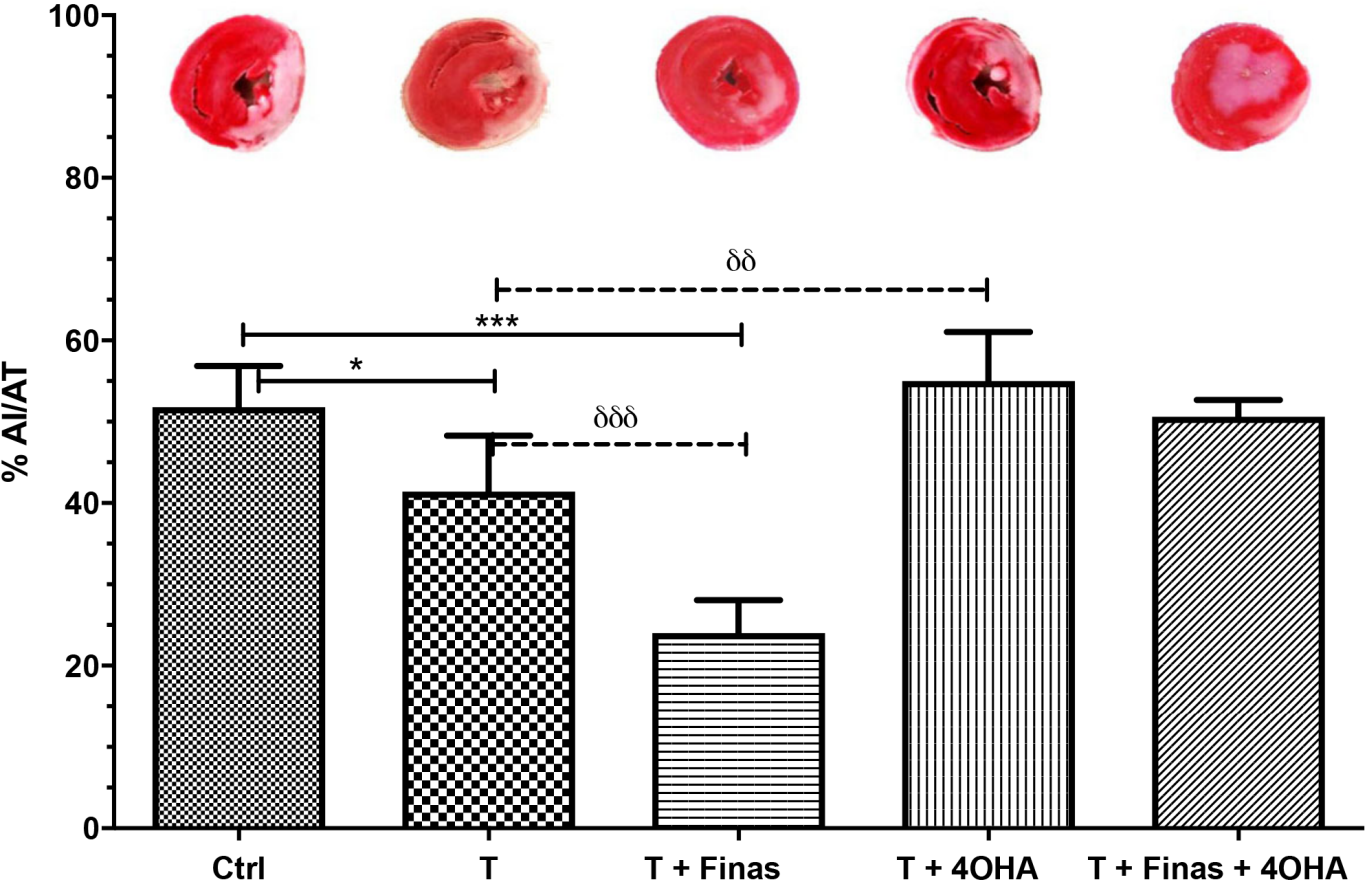
**Effect induced by the inhibition of testosterone metabolism.** Testosterone modifies the percentage of tissue damage by the coronary I/R process in ORX rats. The ORX rats were treated subcutaneously every 72 h for 30 days after cardiac damage induced by the coronary I/R process, with exogenous testosterone (T), Finasteride (Finas), 4-OHA or a combination of both inhibitors. Representative images of heart sections are shown at the top of each bar. Image X4. The data are expressed as the mean±s.e.m. of the percentage of the AI/AT ratio of five hearts per group, *P<0.05, ^δδ^*P*<0.01, ***P<0.005, ^δδδ^*P*<0.001.

In order to evaluate the role of testosterone during I/R, we administered exogenous testosterone to ORX rats. Interestingly, testosterone administration reduced the percentage of damaged heart tissue when compared to the control group (41.4±6.9 versus 51.8±5.1, % AI/AT, respectively, *P*<0.05) (Fig. 1).

**Fig. 2.**
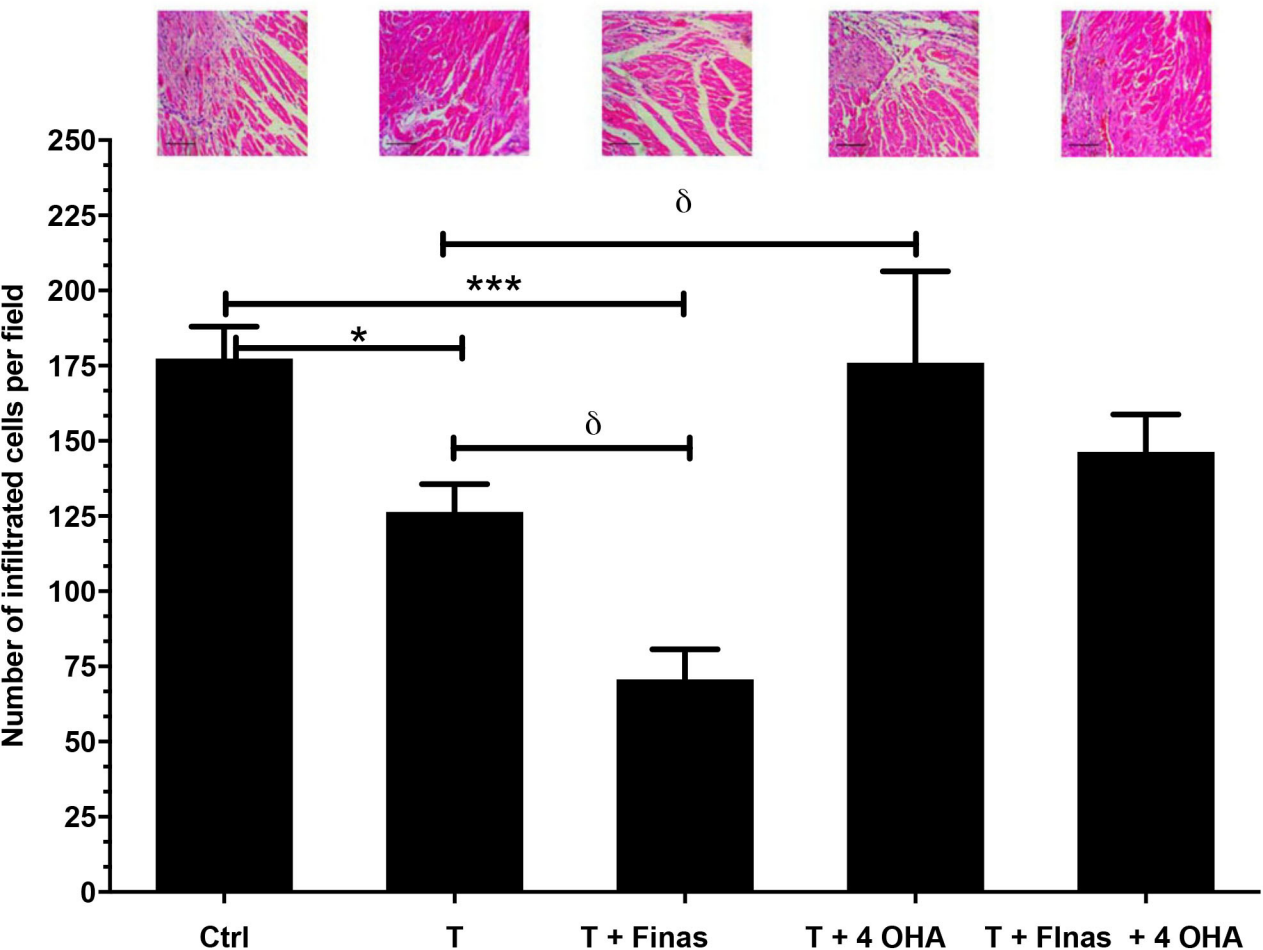
**Quantitative analysis of cellular infiltration (blue spots) in cardiac tissue subjected to coronary I/R in ORX rats.** Exogenous testosterone administration, Finas, 4-OHA or a combination of both inhibitors were administered subcutaneously every 72 h for 30 days after ischemic damage. The analysis was performed in three sections of each heart per group (*n*=5) using the ImageJ program. The data are expressed as the mean±s.e.m. Representative images of cellular infiltration are observed through Hematoxylin-Eosin staining (top panels). Scale bars: 100 μm. *P<0.01, ^δ^*P*<0.05, ***P<0.001.

We evaluated whether the conversion of testosterone into 17β-estradiol or DHT was responsible for the beneficial effects of testosterone on I/R. To do this, we administered 5α-reductase (Finasteride) and/or aromatase (4-OHA) inhibitors. Finasteride administration in testosterone+ORX treated rats resulted in a significant decrease in myocardial damage when compared to both the untreated ORX (51.8±5.1 versus 24±4.1, control versus testosterone+Finas % AI/AT, respectively, *P*<0.001) and the ORX group treated with testosterone (41.4±6.1 versus 24±4.1, testosterone versus testosterone+Finas, respectively, *P*<0.001). The protection induced by testosterone during I/R disappeared when aromatase was inhibited with 4-OHA (41.4±6.9 versus 55±6% AI/AT, respectively, *P*<0.01) (Fig. 2). On the other hand, simultaneous enzymatic inhibition of 5α-reductase and aromatase did not induce significant changes when compared with either the ORX control group or the ORX group administered with testosterone (Fig. 1).

### Histological analysis

We evaluated the cellular infiltration and changes in cardiac tissue architecture 30 days after coronary I/R by Hematoxylin-Eosin staining. Testosterone administration decreased the presence of inflammatory cells in the ORX group compared to the untreated ORX group (*P*<0.05). Finasteride administration resulted in decreased cellular infiltrate. This group presented fibers of a normal size and morphology while edema was scarce. The testosterone+4-OHA group was the most affected, with a loss of continuity and separation of cardiac muscle fibers due to a dense inflammatory infiltrate. Administration of both 4-OHA and Finasteride in testosterone+ORX did not result in statistical differences compared to the untreated control (Fig. 2).

### MMP-3 expression in cardiac tissue

**Fig. 3.**
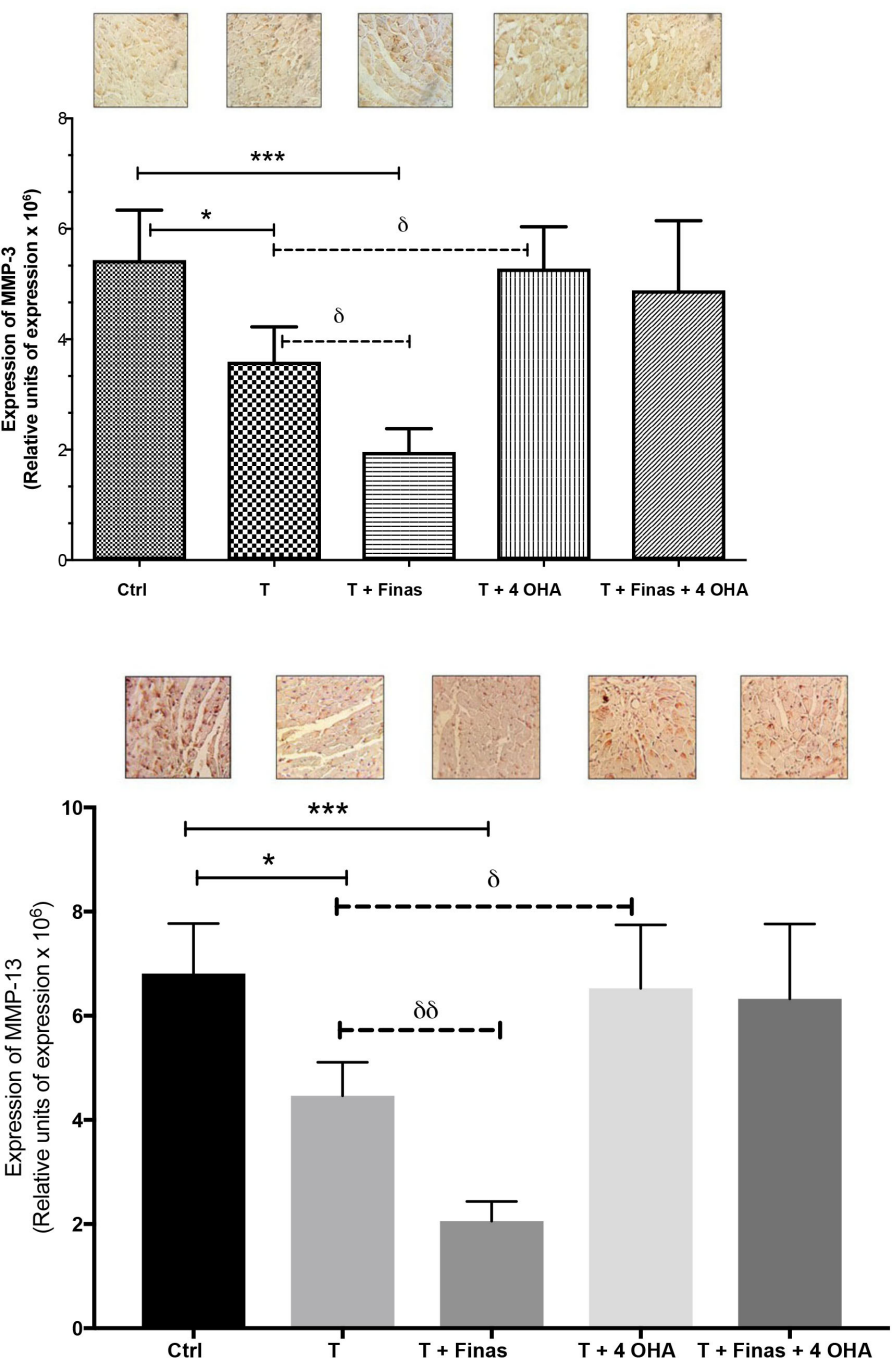
**Quantitative analysis of MMP-3 and MMP-13 expression.** Upper panel: MMP-3 expression in cardiac tissue subjected to coronary I/R in ORX rats. Lower panel: MMP-13 expression in cardiac tissue subjected to coronary I/R in ORX rats. Rats were treated subcutaneously with exogenous testosterone, Finas, and/or 4-OHA every 72 h for 30 days after ischemic damage. The micrographs show representative images of the immunohistochemical analysis performed on five sections of each heart per group (*n*=5). The data are expressed as the mean±s.e.m. *P<0.01, ^δ^*P*<0.05, ***P<0.001, ^δδ^*P*<0.01. Scale bar: 100 μm.

Fig. 3 shows micrographs of myocardial histological sections showing immunoreactive zones for MMP-3 and their respective quantitative analysis by measuring the integrated optical density of the treated groups and the control group. Testosterone administration decreased the expression of MMP-3 when compared to the control group (5.4×10^6^±0.9×10^6^ versus 3.6×10^6^±0.6×10^6^, control versus testosterone, respectively, *P*<0.05) (Fig. 3, upper panel). Administration of testosterone+Finas resulted in decreased MMP-3 expression in contrast to the control group (5.4×10^6^±0.9×10^6^ versus 1.9×10^6^±0.4×10^6^, control versus testosterone+Finas, respectively, *P*≤0.001) and the testosterone group (3.6×10^6^±0.6×10^6^ versus 1.9×10^6^±0.4×10^6^, testosterone versus testosterone+Finas, respectively, *P*≤0.05) (Fig. 3, upper panel). The enzymatic inhibition of aromatase abrogated the effects of testosterone administration on MMP-3 expression (3.6×10^6^±0.6×10^6^ versus 5.3×10^6^±0.8×10^6^, testosterone versus testosterone+4-OHA, respectively, *P*≤0.05). Interestingly, co-administration of the enzymatic inhibitors Finasteride and 4-OHA did not induce significant changes (Fig. 3, upper panel).

### MMP-13 expression in cardiac tissue

Fig. 3 (lower panel) shows micrographs of myocardial histological sections showing immunoreactive zones for MMP-13 and their respective quantitative analysis by measuring the integrated optical density of the treated groups and the control group. The administration of testosterone significantly decreased MMP-13 (6.8×10^6^±1×10^6^ versus 4.5×10^6^±0.6×10^6^, control versus testosterone, respectively, *P*≤0.05). It was also observed that Finasteride administration in the presence of testosterone increased the inhibitory effect on MMP-13 expression (6.8×10^6^±1×10^6^ versus 2×10^6^±0.4×10^6^, control and testosterone+Finas, respectively, *P*≤0.001).

The enzymatic inhibition of 5α-reductase in the presence of testosterone decreased the expression of MMP-13. This reduction was additional to the effects observed in the groups that received testosterone only (4.5×10^6^±0.6×10^6^ versus 2×10^6^±0.4×10^6^, testosterone versus Finas, respectively, *P*≤0.05). In addition, the administration of testosterone and simultaneous inhibition of 4-OHA significantly increased the MMP-13 expression when compared with the testosterone treated group (4.5×10^6^±0.6×10^6^ versus 6.5×10^6^±1.4×10^6^, testosterone versus testosterone+4-OHA, respectively, *P*≤0.05). Finally, simultaneous administration of the enzymatic inhibitors Finasteride and 4-OHA did not induce significant changes.

It has been postulated that testosterone plays a fundamental role in cardiovascular function; however, divergent findings have raised controversy regarding its possible beneficial effects. Several studies have shown cardioprotective effects of testosterone. For example, Liu et al. (2006), observed that testosterone administration contributes to reducing infarct size in ventricular myocytes isolated from male rats subjected to ischemic preconditioning and gonadectomy (Liu et al., 2006). Er et al. (2004) demonstrated that testosterone was directly cytoprotective in the myocardium through ATP sensitive K+ channel activation in the inner mitochondrial membrane (Er et al., 2004). In 2003, Callies and colleagues showed in a cardiac I/R model (Langendorff system) that testosterone improved cardiac function recovery in gonadectomized rats (Callies et al., 2003).

In contrast, deleterious effects of testosterone have also been reported. For example, testosterone administration induced exacerbated inflammatory cytokines production, p38 MAPK pathway activation, apoptotic signaling and androgenic receptors blockade with flutamide, or castration improved myocardial function after I/R (Wang et al., 2005; Vannay et al., 2009). In animal models, supraphysiological concentrations of anabolic steroids induced deleterious cardiac remodeling and impaired cardiac function (Rocha et al., 2007). Clinical studies support the evidence that anabolic androgenic steroids increase the content of myocardial collagen. This effect contributes to a deterioration of systolic function (Fanton et al., 2009).

On the other hand, in general, the heart is considered only a testosterone target tissue, meaning that circulating testosterone that originated in gonads needs to reach and interact with androgenic receptors at the myocardial level in order to induce its effects; however, this does not seem to be the main/only phenomena since at the myocardial level testosterone can be converted into DHT or estradiol by the competing enzymes 5α-reductase and aromatase, respectively (Regouat et al., 2018). Interestingly, the role of these enzymes' activities in the absence (as in gonadectomized animals) or in states of decreased circulating testosterone is poorly understood. We showed that ORX rats had a significant increase in 5α-reductase expression without changes in aromatase expression, our results suggested a cross-modulation of the activities of these enzymes since inhibition of their activities induced divergent effects in acute I/R-induced myocardial damage (Regouat et al., 2018).

In the present work, testosterone administration in ORX rats reduced cardiac remodeling (scar tissue size) (*P*<0.05) after 30 days of I/R-induced myocardial damage. In addition, chronic 5α-reductase inhibition by Finasteride resulted in a significant reduction (*P*<0.001) in cardiac pathological remodeling (scar tissue size and inflammatory infiltrates) (*P*<0.05). In contrast, aromatase inhibition by 4-OHA increased pathological remodeling (*P*<0.01), inducing disseminated lymphocytic infiltrate in perivascular and interstitial areas, enlarged cardiomyocytes, and intra and extracellular edema.

These results suggest that, in the absence of gonadal testosterone, the balance between 5α-reductase and aromatase enzymatic activity plays a role in the effects attributed to testosterone, favoring protection when aromatization predominates and damage when 5α-reduction occurs. This idea is reinforced when we analyze the affinity constants (Km Michaelis-Menten). Aromatase affinity constant is 1.83 nM while 5α-reductase is 3.35 nM. Thus, it is more feasible (1.53 times) the aromatization of testosterone than 5α-reduction (Lakshman et al., 2010). Our results show that the simultaneous chronic inhibition of both enzymes (Finas+4-OHA) induced no changes in cardiac damage as compared to controls, suggesting also that testosterone by itself has no direct effect being necessary its 5α-reduction or aromatization to induce effects.

On the other hand, several studies have shown that MMPs play a fundamental role in several processes of cardiovascular diseases. These include atherosclerotic plaque rupture, (Galis and Khatri, 2002) acute myocardial infarction, (Webb et al., 2006) aneurysm and left ventricular rupture (Carmeliet, 2000), ventricular remodeling (Zile et al., 2011; Spinale, 2007) and age-dependent changes in the left ventricular structure (Lindsey et al., 2005). Yet, during ventricular remodeling, the effects of testosterone metabolism on MMP-3 and MMP-13 have not been fully explored. MMP-3 is a protease capable of degrade proteoglycans, fibronectin, laminin and type IV collagen and is able to activate other MMPs. MMP-3 may play a regulatory role during myocardial remodeling (Mukherjee et al., 2005). High MMP-3 levels are associated with left ventricular dysfunction and adverse left ventricular remodeling after acute myocardial infarction (AMI) (Kelly et al., 2008). Myocardial MMP-3 expression is elevated early after experimental AMI and sustained for several days (Romanic et al., 2001). In men, MMP-3 expression levels are higher 3 months after AMI than 48 h after AMI (Cavusoglu et al., 2016). On the other hand, MMP-13 degrades fibrillar collagen type I and III (Vincenti et al., 1998), and it is expressed at moderate levels in the healthy heart. Although MMP-13 expression increases under cardiac pathological conditions (Li et al., 2000; Sakata et al., 2004), its role in pathological cardiac remodeling remains unknown. After an ischemic event and in patients with chronic heart failure MMP-13 expression and activity remains elevated for months or years. This results in pathological cardiac remodeling progression (Jaffré et al., 2012).

The pharmacological inhibition of metalloproteases in animals with AMI attenuates the remodeling process preserving cardiac function (Spinale, 2002; Rohde et al., 1999). These data suggest an important participation of metalloproteases in the remodeling and ventricular dysfunction, secondary to AMI. Our results show that exogenous testosterone administration to ORX rats during coronary I/R resulted in decreased inflammatory infiltrates (*P*<0.05) and reduced MMP-3 and MMP-13 expression (*P*<0.05, for both cases). Interestingly, Finasteride administration (Ki 2.1 μM) (Drury et al., 2009) resulted in a greater decrease in scar tissue (*P*<0.05), along with a significant decrease in MMP-3 (*P*<0.05) and MMP-13 expression (*P*<0.01).

These observations suggest that DHT is responsible for the detrimental effects on cardiac function/morphology induced by testosterone. This is in agreement with our previous reports showing DHT as an inductor of myocardial damage (Regouat et al., 2018).

In contrast, 4-OHA (Ki 3.28 μM) administration (Block et al., 1996) increased MMP-3 (*P*<0.05) and MMP-13 (*P*<0.01) expression. These results suggest that testosterone aromatization is associated with cardioprotection.

In conclusion, testosterone administration significantly decreased cardiac remodeling in ORX rats after 30 days of I/R-induced myocardial damage. In addition, testosterone aromatization was necessary to preserve its cardioprotective effect. The expression levels of MMP-3 and MMP-13 were downregulated when 5α-reduction was inhibited. Our results suggest that testosterone metabolism deserves more detailed attention when testosterone effects are explored at the cardiovascular level.

In this study we cannot define whether the changes in MMPs expression were a consequence of decreased tissue damage or were caused by enzymatic inhibition. Regardless of this, the beneficial and significant changes found contribute to the understanding of the complex paradigm of testosterone 5α-reduction or aromatization on cardiac remodeling.

## MATERIAL AND METHODS

### Ethical considerations

All procedures were performed in accordance with the Guide for the Care and Use of Laboratory Animals approved by the National Institutes of Health of the United States and by the Official Mexican Standard (NOM-062-ZOO-1999).

### Orchiectomy

55 male Wistar rats were used, of which 50 were bilaterally ORX according to Svensson et al. (2000). In brief, rats (200–250 g) were anesthetized with sodium pentobarbital [60 mg/kg, intraperitoneally (i.p.); Abbott Laboratory, Chicago, IL, USA]. An incision (2 cm) was made in the midline of the scrotum to expose the testicle. The vas deferens and main arteries and veins were isolated, ligated and excised. This allowed the elimination of each testicle and epididymis (Golden et al., 2003). The animals were allowed to recover in separate cages for 15 days. Animals were kept at the university bioterium with food and water *ad libitum*.

Total duration of castration before I/R was 6 weeks.

### Ischemia/coronary reperfusion model

The ORX rats (*n*=50) and the control group (no orchiectomy) (*n*=5) were anesthetized with pentobarbital (0.1 m/100 g, i.p.). The rats were ventilated by a tracheal cannula with air enriched with oxygen using a positive pressure ventilator (Ugo basile, MODEL 7025). An electric blanket was used to maintain the rat temperature at 37°C. Left thoracotomy (4–5 left intercostal space) was performed and the pericardium was opened to expose the heart. The anterior descending coronary artery was ligated approximately 1 cm below the atrial appendage with a sterile silk suture (6-0). To avoid coronary artery damage, a 3–5 mm Teflon tube was placed between the artery and the silk suture. The blood flow occlusion was maintained for 1 h verified by color change, subsequently, the Teflon tube was removed to allow reperfusion and the thorax was closed.

Animals were randomly assigned in the following groups: (1) vehicle control (*n*=10), (2) testosterone (testosterone enanthate) (*n*=10), (3) testosterone and Finasteride (*n*=10), (4) testosterone and 4-hydroxyandrostenedione (*n*=10), (5) testosterone, Finasteride and 4-hydroxyandrostenedione (*n*=10). A group of not ORX rats with I/R (*n*=5) was also included. Unless otherwise indicated, the reagents were purchased from Sigma-Aldrich. The treatments were administered subcutaneously every 72 h for 30 days. The doses used were previously determined (data not shown): testosterone (346 ng/kg), Finasteride (Finas, 1.8 μg/kg) and 4-OHA (1.8 μg/kg).

At the end of the treatments, five rats per group were randomly chosen for cardiac viability analysis. The remaining five rats were used for histological and immunohistochemical analysis.

### Myocardial damage evaluation

The heart was removed under anesthesia, administration of 0.5 ml of 154 mM KCl was used to stop the heart during diastole, and then the atria and larger vessels were eliminated. The ventricles were kept at −20°C for 2 h and processed into 2-mm-thick cross-sections. Sections were incubated in a 1% triphenyl tetrazolium solution for 20 min at 37°C, followed by immersion in 4% paraformaldehyde (pH 7.4). The images were analyzed in ImageJ 1.50i Software (NIH, USA) in a double-blind condition. The cardiac damage was calculated by determining the relationship between the infarcted area (unstained) and the total area (IA/AT) (Downey).

### Histological analysis

The isolated hearts were immediately sectioned into four equal-sized parts, incubated in 4% paraformaldehyde and embedded in paraffin (Paraplast^®^).

### Hematoxylin-Eosin staining

Sections of 5 μm were stained with Hematoxylin-Eosin for histopathological evaluation. Images were obtained using a Nikon Plan Fluor optical microscope and a digital camera. The quantitative analysis of inflammatory cell infiltration was performed using ImageJ software. Three infiltrated sections were analyzed for each heart and treatment group.

### Immunohistochemistry

Slices (5 μm) were dewaxed in xylene to remove paraffin and rehydrated with graded alcohol (100%, 95%, 90%, 80% and 70%) and finally 100 mM phosphate buffer, pH=7.4, at room temperature on three consecutive occasions for 5 min each. Then the slices were incubated in phosphate buffer 2% albumin for 1 h. Subsequently, the sections were incubated with primary antibody against MMP-3 (sc-6839 goat polyclonal antibody, Santa Cruz Biotechnology, dilution 1:100) or with primary antibody against MMP-13 (sc-30073, rabbit polyclonal antibody, Santa Cruz Biotechnology, dilution 1:100). Next, the sections were washed with 100 mM phosphate buffer solution, pH 7.4, at room temperature on three consecutive occasions for 5 min. Incubation was carried out with secondary antibodies coupled to horseradish peroxidase (sc-3837 anti-goat IgG and sc-3851 anti-rabbit IgG, Santa Cruz Biotechnology, respectively, 1 h at room temperature). The slices were washed and incubated in diaminobenzidine solution (5 mg diaminobenzidine and 5 μl peroxide 30%) in 10 ml of 100 mM phosphate buffer, pH 7.4, at room temperature for 10 min. The sections were counterstained with Hematoxylin-Eosin.

Images were acquired by using a Nikon Plan Fluor microscope and evaluated with the ImageJ digital analyzer (1.50i) (https://imagej.nih.gov/ij/). Seven sections of each heart were analyzed.

### Statistical analysis

The data are presented as the mean±standard deviation of the mean (s.d.). For the graphic representation and the statistical analysis of the results, the GraphPad Prism 6.01 program was used. We used the D'Agostino–Pearson test for normality**.** The statistical significance between the experimental groups was determined with an analysis of variance (ANOVA). The *F*-test was used to perform comparisons. The *P*-value <0.05 was considered statistically significant.
